# Comparison of Recurrence Rate and Outcomes of Pterygium Surgery with Inferior Versus Superior Rotational Conjunctival Flap: A Randomized Clinical Trial

**DOI:** 10.18502/jovr.v21.17916

**Published:** 2026-08-21

**Authors:** Hassan Behboudi, Mitra Akbari, Reza Soltani Moghadam, Hamed Molaei, Ehsan Kazemnezhad Leili

**Affiliations:** Eye Research Center, Department of Ophthalmology, Amiralmomenin Hospital, School of Medicine, Guilan University of Medical Sciences, Rasht, Iran

**Keywords:** Pterygium Surgery, Recurrence, Rotational Conjunctival Flap

## Abstract

**Purpose:**

Limited knowledge exists about the recurrence and complications of rotational flaps harvested from the inferior bulbar conjunctiva in pterygium surgery. This study aimed to investigate the outcomes and recurrence rate of pterygium surgery using inferior rotational conjunctival flap (IRCF) compared with superior rotational conjunctival flap (SRCF) surgery.

**Methods:**

This randomized clinical trial was performed on 82 candidates for pterygium surgery (primary pterygium with horizontal extension > 3 mm). The patients had been admitted to Amiralmomenin Hospital (Rasht, Iran) in 2023-2024 and were randomized to “group A” (IRCF surgery) and “group B” (SRCF surgery) using a single-blind method. They were visited on days 1, 3, 7, 30, 90, and 180 post-surgery, and their best corrected visual acuity (BCVA), clinical inflammation, complications, and recurrence were recorded and compared using SPSS version 26.

**Results:**

Recurrence was observed in two patients in each group: in group A, one case in the third month and another in the sixth month post-surgery; and in group B, two cases in the third month post-surgery. Frequency of corneal epithelial defect healing, frequency of complications, inflammation severity, BCVA, and recurrence rate did not differ between the two groups at any follow-up visits (*P* > 0.05). There was no statistically significant difference between the two groups in terms of recurrence rates, categorized by pterygium size and age, at any time point (*P* > 0.05).

**Conclusion:**

Pterygium surgery with IRCF can be a safe and effective method for treating primary pterygium, especially when potential glaucoma filtration surgery is considered or when a proper superior bulbar conjunctiva is not available. Further multicenter prospective trials are needed to confirm the benefits of this technique.

##  INTRODUCTION

Pterygium is a triangular, wing-shaped degenerative disease of the conjunctiva extending onto the cornea. It is usually located in the interpalpebral zone and presents with redness, burning, dryness, tearing, and decreased vision.^[1,2]^ It has a relatively high prevalence of about 12%, which increases to 19.5% in the elderly ( > 80 years). Patients at a higher risk include those exposed to ultraviolet radiation, those living in tropical and rural areas, and those not using sunglasses; however, the exact pathophysiology of the disease is still unknown.^[3]^

For patients seeking symptomatic, optical, or cosmetic improvement, surgery is performed using a variety of techniques such as conjunctival autograft, primary closure of the conjunctiva, limbal-conjunctival autograft, amniotic membrane graft, and conjunctival rotation autograft. The primary treatment is conservative and involves the use of lubricants and sunglasses.^[4]^ Research on the advantages of various surgical procedures is still ongoing, with a primary focus on recurrence rate, postoperative complications, and surgical time.^[5]^ Surgical factors, such as excessive suturing, insufficient conjunctival graft size, thick conjunctiva, and graft retraction,^[6]^ as well as pterygium size and increased vascularity^[7]^ are linked to a higher chance of recurrence following surgery. To lower the risk of surgery, physicians advise adjuvant drugs such as corticosteroids, mitomycin C, 5-fluorouracil, and anti-vascular endothelial growth factors.^[6,8]^ Still, recurrence is considerably high ( > 50%) after simple surgical excision with the bare sclera technique.^[9]^

Conjunctival rotational flap (CRF) is considered to have low rates of recurrence, graft displacement, and necrosis.^[8,10]^ Because the conjunctival flap in the traditional version of CRF is taken from the upper bulbar conjunctiva, individuals with superior conjunctival scarring or those suspected of developing glaucoma should not have this type of pterygium surgery, but may require subsequent filtering surgery. Another surgical method that has a profile comparable to the traditional technique involves harvesting the inferior bulbar conjunctiva.^[11,12]^

Considering limited knowledge about the recurrence and complications of grafts harvested from the inferior bulbar conjunctiva, in the present study, we aimed to investigate the complications and recurrence of pterygium surgery using an inferior rotational conjunctival flap (IRCF) and compare the results with superior rotational conjunctival flap (SRCF) surgery in a randomized clinical trial.

##  METHODS

### Study Design

A controlled, parallel-group, single-blind, randomized trial was performed on 82 candidates for pterygium surgery who were referred to the Amiralmomenin Hospital (Rasht, Iran) during 2023-2024. The protocol was submitted to the Iranian Registry of Clinical Trials at https://irct.behdasht.gov.ir/ for registration (code: IRCT20160919029871N6) and approved by the Ethics Committee of Guilan University of Medical Sciences (code: IR.GUMS.REC.1401.448).

### Inclusion Criteria 

Pterygium surgery was recommended for patients whose primary pterygium measured more than 3 mm and extended horizontally into the cornea.

### Exclusion Criteria 

The study excluded patients with a history of recurrent pterygium or pseudo-pterygium, those who were pregnant or nursing, those who had systemic diseases like diabetes mellitus or heart problems, those who had ocular surface disorders like keratitis, conjunctivitis, or extremely dry eyes, those who had a history of herpetic keratitis, and those who had undergone eye surgery within the previous 6 months. Patients were excluded from the study if the treatment protocol changed for any reason, such as when they did not attend the follow-ups, when medication orders were not correctly filled, or when medication was not stored properly. Furthermore, patients were excluded from the trial if the pterygium base in the limbus area measured > 8 mm.

### Sample Size and Group Allocations 

We used the Power Analysis & Sample Size (PASS) software to determine the appropriate sample size based on power analysis and accordingly assigned 40 individuals to each group. This was achieved by considering a 95% CI, 80% power, and a risk of 0.096, following the study by Yeung et al.^[13]^ Patients who met the inclusion criteria were enrolled and divided into two groups using random quadruple blocks as follows:

• Group A, who underwent IRCF surgery
• Group B, who underwent SRCF surgery

At the Ophthalmology Research Center, the block sequence list was stored in sealed envelopes. With the approval of the university ethics committee, the envelope was unsealed at the start of the trial, and patients were sequentially allocated to either group A or B. The patients were blinded to group allocation.

### Perioperative and Pterygium Operation

Before surgery, the cornea surgeon visited and examined the patients and recorded the results of the anterior segment examination, pterygium size (from the pterygium tip to its base on the limbus), and grading—categorized based on the Tan classification system into T1 (atrophic), T2 (moderate), and T3 (fleshy). All procedures were performed by a cornea surgeon (MA) using the pterygium surgery with rotational conjunctival flap (RCF) method. To cover the sclera, a supranasal conjunctival flap was used in one group (group B) and an infranasal conjunctival flap in the other group (group A). In all cases, Nylon 10.0 was used for suturing the conjunctival flap to the episclera and around the conjunctiva with an interrupted suturing technique. Additionally, mitomycin C was used for 90 seconds intraoperatively for all patients. The day after surgery, topical antibiotics (chloramphenicol 0.5%) and topical steroid (betamethasone 0.1%) were prescribed to be taken four times a day. The antibiotic was discontinued after complete healing of the corneal epithelium, and the steroid dose was tapered within 2 months and then stopped. The time of suture removal in all cases was 1 month after the operation.

### Follow-up 

Patients' age and sex were recorded, and they were recommended to refer to the ophthalmology clinic on days 1, 3, and 7, as well as at 1, 3, and 6 months after surgery. The best corrected visual acuity (BCVA), intraocular pressure (IOP), clinical inflammation of the pterygium surgery site, the length of time taken to repair the corneal epithelial defect, and any potential complications post operation were all noted by the ophthalmologist during each visit. Recurrence rate was the study's main endpoint, whereas corneal epithelial defect healing time, surgical complications, and BCVA were its secondary outcomes.

#### Primary outcome

The recurrence was defined as grades 2 and 3, following the classification suggested by Prabhasawat et al.^[14]^ Patients were categorized into grades 0 through 3 based on slit-lamp examination of the anterior segment. Grade 0 was described as no recurrence, while grade 1 was defined as the presence of modest sclera vascularity around the resected region that was not connected to fibrosis. Fibrovascular growth limited to the sclera in the resected region was referred to as grade 2, and fibrovascular proliferation extending outside the limbus was classified as grade 3.

#### Secondary outcomes

BCVA was measured by logMAR (Logarithm of the Minimum Angle of Resolution). Based on hyperemia at the surgical site, clinical grading for postoperative inflammation was assessed at 1 and 3 months postoperatively. Grade 1 denoted no redness or pink color, grade 2 denoted scattered patches of mild redness, and grade 3 denoted diffuse and noticeable redness.^[15]^ Any postoperative complications were recorded and included Tenon cyst, granulation tissue, persistent corneal epithelial defect, delayed conjunctiva healing, graft necrosis, scleral thinning, Dellen formation, and other minor complications such as foreign body sensation, tearing, and burning.

### Statistical Analysis

Once the data-gathering process was completed, data were entered into SPSS version 26.0 (IBM Corp., Armonk, NY, USA). The recurrence was described using frequency and 95% confidence interval (CI). Either Fisher's exact test or Pearson's chi-square test was used to compare the variables between the groups. Levene's test was used to assess equality of variances, and the Kolmogorov-Smirnov test was used to assess the normal distribution of numeric variables. Additionally, the independent samples *t*-test was employed for variables with a normal distribution and the Mann-Whitney U test for variables without a normal distribution in order to compare the numerical variables between the two groups. Variables with and without a normal distribution were compared using one-way ANOVA or the Kruskal-Wallis test, respectively. A significance threshold of < 0.05 was considered.

##  RESULTS

### Baseline Characteristics

**Figure 1:**
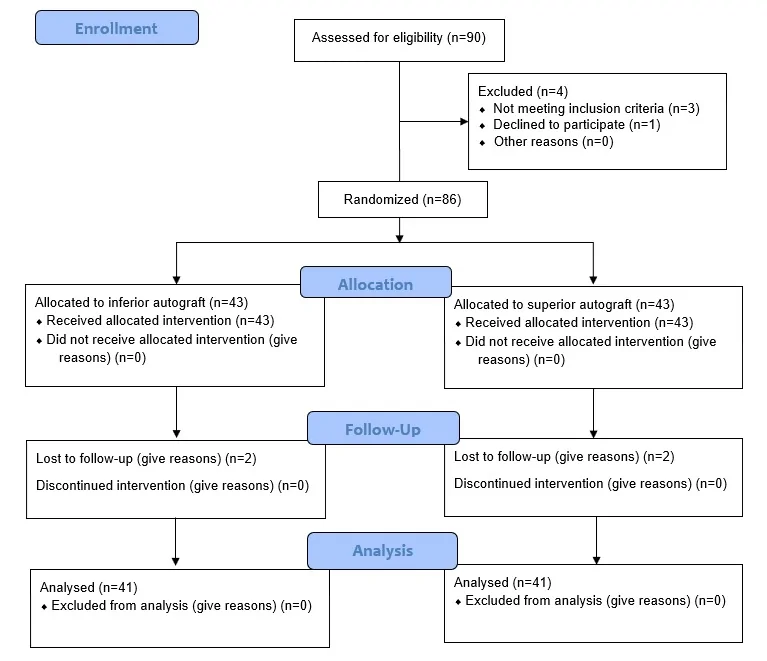
Flow diagram of participants' screening and enrollment for this study.

The trial was completed by 82 participants in total (41 in each group) [Figure 1]. There was no significant difference in the mean age of the patients or the distribution of sexes between the two groups (*P* > 0.05) [Table 1]. Additionally, there was no difference between the two groups in baseline clinical features such as the mean size of the pterygium, IOP, Tan categories, and right or left eye (*P* > 0.05) [Table 1].

**Table 1:** Comparing baseline variables between the two study groups

**Variable**		**Group A**	**Group B**	***P*** **-value**
Sex, *N* (%)	Male	24 (48)	26 (52)	0.651 ${}^{*}$
	Female	17 (53.1)	15 (46.9)	
Age (yr), mean ** ± ** SD		58.44 ± 12.38	59.68 ± 11.54	0.639 ${}^{†}$
Pterygium size (mm), mean ** ± ** SD		3.72 ± 0.55	3.81 ± 0.56	0.520 ${}^{†}$
Intraocular pressure (mmHg), mean ** ± ** SD		13.61 ± 2.22	14.03 ± 2.12	0.421 ${}^{†}$
Tan category	Tan 1	0	0	0.616 ${}^{*}$
	Tan 2	40 (51.3)	38 (48.7)	
	Tan 3	1 (25)	3 (75)	
OD-OS ${}^{\#}$ , *N* (%)	Right eye (OD)	21 (56.8)	16 (43.2)	0.267 ${}^{*}$
	Left eye (OS)	20 (44.4)	25 (55.6)	
${}^{*}$ The results of the chi-square test; ${}^{†}$ the result of the independent samples *t* test. ${}^{\#}$ OD, oculus dexter, OS, oculus sinister.				

### Corneal Epithelial Healing

The results of the postoperative measurements are shown in Table 2. The frequency of corneal epithelial healing in Table 2 shows the significant effect of time in each group; however, the groups were not different at any of the time points (*P* > 0.05). The pairwise comparisons showed a significant difference in each group between days 1 and 3 (*P *= 0.004 in group A and *P = *0.021 in group B); additionally, the values on days 1 and 3 were different from those on day 7, as well as months 1, 3, and 6 (all *Ps* < 0.001), while the values at the other follow-ups were not different (all *Ps* > 0.999).

### Postoperative Complications

There were no postoperative complications in the study population, except for one case of granulation tissue in group B. No difference was observed between the two groups or within each group (*P* > 0.05) [Table 2]. The eye burning was not different between the two study groups at any follow-up visits (*P* > 0.05). The burning sensation was based solely on subjective evaluation.

**Table 2:** Comparing postoperative variables between the two study groups

**Variable**	**Follow-up **	**Group A**	**Group B**	***P*** -value ${}^{*}$
Corneal epithelial defect	Day 1	41 (100)	41 (100)	> 0.009
	Day 3	25 (61)	16 (39)	0.647
	Day 7	5 (12.2)	2 (4.9)	0.236
	Month 1	0	0	> 0.009
	Month 3	0	0	> 0.009
	Month 6	0	0	> 0.009
	*P*-value ${}^{†}$	< 0.001	< 0.001	
Postoperative complications	Day 1	0	0	> 0.999
	Day 3	0	0	> 0.999
	Day 7	0	1 (2.4)	> 0.999
	Month 1	0	1 (2.4)	> 0.999
	Month 3	0	1 (2.4)	> 0.999
	Month 6	0	0	
	*P*-value ${}^{†}$	> 0.999	0.629	
${}^{*}$ The results of Pearson's chi-square test; ${}^{†}$ the result of Cochran's Q test.				

### Postoperative Inflammation Grades

Comparing the results of inflammation grade, as shown in Table 3, indicated no difference between the two study groups in the frequencies of grade 1 (G1), G2, and G3 at any follow-ups (*P* > 0.05), however, the effect of time was significant in each group. Pairwise comparison showed that, within each group, measurements on day 3 were not different from day 1 (*P* > 0.999 for both groups), and day 7 was not different from days 1 and 3 (both *Ps* > 0.999 in group A and 0.129 and 0.582, respectively, in group B). However, months 1, 3, and 6 were different from days 1, 3, and 7 after the surgery (all *Ps* < 0.001) but not from the other months (all *Ps* > 0.999).

### Recurrence

The recurrence rate did not differ between the groups at any of the follow-ups (*P* > 0.05) and within each group at different follow-ups (*P* > 0.05). Moreover, the recurrence grading was not different based on sex, age ( ≤ 59 vs > 59 years), or pterygium size (3 vs 3.5, 4 vs 4.5, and 5 mm; all *Ps* > 0.999). Only two patients in each group experienced the recurrence of pterygium (about 5%); the two cases in the IRCF group occurred at months 3 and 6, both of whom were grade 2. On the other hand, the two cases in the SRCF group occurred at month 3, and they were grade 3 recurrences. The recurrence rate, categorized by pterygium size, sex, and age, did not differ between the groups at any of the follow-ups.

**Table 3:** Comparing the inflammation grading between the two study groups

**Variable**	**Follow-up **	**Grading**	**Group A**	**Group B**	***P*** -value ${}^{*}$
Inflammation grade	Day 1	G1 ${}^{\#}$	0	0	0.775
		G2	34 (82.9)	33 (80.5)	
		G3	7 (17.1)	8 (19.5)	
	Day 3	G1	0	0	> 0.999
		G2	40 (97.6)	39 (95.1)	
		G3	1 (2.4)	2 (4.9)	
	Day 7	G1	6 (14)	11 (26.8)	0.429
		G2	34 (82.9)	29 (70.7)	
		G3	1 (2.4)	1 (2.4)	
	Month 1	G1	35 (85.4)	38 (92.7)	0.482
		G2	6 (14.6)	3 (7.3)	
		G3	0	0	
	*P*-value ${}^{†}$		< 0.001	< 0.001	
${}^{*}$ The results of Mann-Whitney U test; ${}^{†}$ the result of Friedman test; ${}^{\#}$ Grading.					

### Visual Acuity

**Figure 2:**
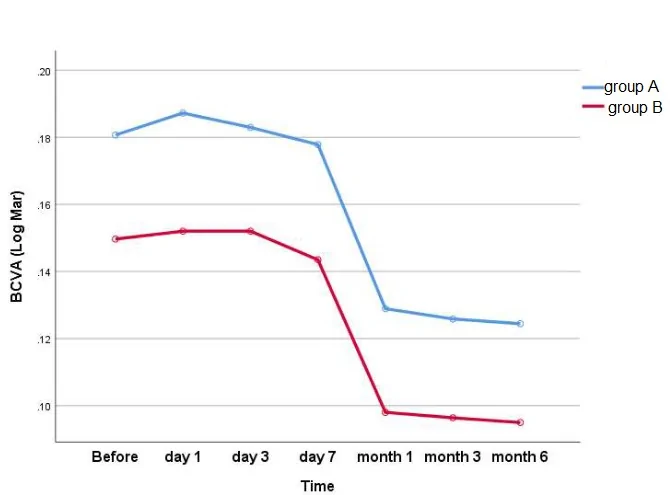
Trend of changes in best-corrected visual acuity (logMAR).

The mean BCVA was not different between the two groups before the surgery (*P *= 0.400), on day 1 (*P *= 0.305), day 3 (*P *= 0.362), day 7 (*P *= 0.294), month 1 (*P *= 0.254), month 3 (*P *= 0.272), and month 6 (*P *= 0.271). The effect of group was not significant (*P *= 0.292), while the effect of time was significant in each group (*P* < 0.001) with an effect size of 0.353. The trend of changes in BCVA is shown in Figure 2.

##  DISCUSSION

The present study compared the postoperative outcomes of using the inferior versus SRCF in the surgery for pterygium. Previous studies have focused on the comparison of pterygium surgery with superior versus inferior conjunctival autograft techniques, while we compared, for the first time, the postoperative outcomes of pterygium surgery with IRCF versus SRCF techniques.

The results of comparison between two random groups with matched demographic (age/sex) and baseline clinical (mean size of pterygium, IOP, Tan categories, and right versus left eye) characteristics showed that the rate of recurrence, corneal epithelial defect healing time, burning sensation, frequency of complications, severity of inflammation, and postoperative BCVA were similar at 6-month follow-up. These results suggest that the inferior bulbar conjunctival flap is an appropriate alternative to the superior conjunctival flap in pterygium surgery, especially when the superior conjunctiva is scarred or not available for any other reason, or in patients with glaucoma who are candidates for filtration surgery or have undergone glaucoma surgery before.

Only two patients in each group experienced recurrence (about 5%); the two cases in the IRCF group occurred at months 3 and 6, and the two cases in the SRCF group occurred 3 months after the surgery. The recurrence rate, categorized by pterygium size, sex, and age, did not differ between the groups at any of the follow-ups. These results are in line with those reported in a meta-analysis of four randomized clinical trials—comprising 234 eyes in inferior conjunctival autograft and 204 eyes in superior conjunctival autograft—which found no difference in the recurrence rate of the two methods.^[16]^ All of the four studies included in the meta-analysis reported the same results, indicating a similar recurrence rate between the two methods. The overall recurrence rate in these randomized clinical trials is also close to that reported in the present study. Celeva et al reported the recurrence rate of 3/40 (7.5%) for inferior conjunctival autograft and 5/40 (10.2%) for superior conjunctival autograft, with a mean follow-up of 16.6 and 15.5 months, respectively. The surgical procedure in this randomized clinical trial in Macedonia involved using 8-0 vicryl suture.^[17]^ Yeung et al included a smaller number of eyes in each group and reported one case of recurrence in each group (4%) within > 6 months of follow-up using fibrin glue,^[13]^ which is close to the rate found in the present study. In a study by Upadhyaya and Savur, no recurrence was observed in any of the groups within the follow-up of 38-42 days.^[18]^ Considering that most cases of pterygium recurrence occur within 3 to 6 months after surgery,^[6]^ our follow-up period was long enough.

Chen et al reported recurrence in 2/40 (5%) of patients undergoing inferior conjunctival autograft and 3/40 (7.5%) in those undergoing superior conjunctival autograft within 12 months of follow-up using an electrocautery pen.^[19]^ In another study in China, using 10-0 nylon sutures, Hu and Li reported about 5% recurrence in both study groups at 12-month follow-up.^[20]^ These two Chinese studies^[19,20]^ included limbal cells in their conjunctival autograft, which is sometimes reported to be more effective,^[21,22]^ although others do not consider limbal-conjunctival graft superior to conjunctival autograft^[23,24,25]^ and regard limbal damage as an additional risk. It is believed that the full closure of the excision site with relatively normal conjunctival tissue prevents the remaining aberrant conjunctival and episcleral tissues from proliferating and advancing toward the limbus, which accounts for the low recurrence rate in both groups.^[26]^

Regarding the secondary outcome, we measured epithelial defect healing time in the present study, which became 0 in both groups after 1 month, with no difference between the study groups. In the study by Chen et al, patients were visited on days 1, 2, 3, 5, 7, and 14 and at 1, 3, 6, and 12 months after the surgery. Besides, the results of fluorescein staining showed a complete corneal epithelial healing time of 3.1 ± 0.5 days in the group undergoing inferior conjunctival autograft and 3.3 ± 0.6 days in the group undergoing superior conjunctival autograft, indicating no significant difference between the two groups.^[19]^ These results also show no difference in epithelial healing time between inferior and superior autografts, which is consistent with the results of the present study.

Eye burning and inflammatory grading were two additional secondary outcomes that were assessed. Both had high frequencies on days 1, 3, and 7, and gradually improved in both groups, with no discernible differences between them. These findings are consistent with those of Yeung et al, who observed no differences between the groups in terms of mean surgery time, visual analog scale pain score, or amount of pain relief medication used.^[13]^ Additionally, Upadhyaya and Savur showed no difference in analgesic tablets required, pain, pricking sensation, foreign body sensation, and photophobia between the two groups using inferior or superior conjunctival limbal autograft.^[18]^ However, these results are contrary to those reported by Chen et al, who reported less pain, less epiphoria, and less foreign body sensation in the group undergoing inferior conjunctival autograft on days 3 and 5 after surgery, with no serious complications in any of the groups.^[19]^ Furthermore, Kazancı et al reported a higher mean score of visual analog scale in the superior graft group (6.2 ± 1.3) compared with the inferior graft group (4.7 ± 1.88) on day 1, but it lost significance on postoperative day 7.^[27]^ The patients' ocular pain levels were evaluated using the visual analog scale at day 1 and week 1 after surgery. The visual analog scale is a pain measurement tool consisting of a linear scale between 0 and 10, where 0 indicates no pain, and 10 indicates the worst pain imaginable.^[27]^ This may be connected to the effect of blinking-induced friction over the superior bulbar conjunctiva. Given the upper lid's greater range of motion, this could lead to increased ocular surface inflammation and tear film instability in the early postoperative period, which could prolong corneal epithelial healing in patients undergoing superior conjunctival graft and increase their level of discomfort.^[28]^ However, Kazancı et al observed no difference between the study groups in epithelial cell or mucin staining or other cytological changes postoperatively, although the group undergoing inferior conjunctival autograft had more goblet cells in their conjunctiva before and after the operation. They also showed that corneal and conjunctival staining, tear-film break-up time, and Schirmer tests were similar between the study groups.^[27]^ A definite conclusion cannot be made because of the scant evidence available in the current literature.

Postoperative complications were another outcome assessed in this trial. The findings indicated that there was just one instance of granulation tissue in the SRCF group and that there was no discernible difference between the two groups. These findings, which show no serious problems in any of the groups, are in line with what Chen et al described.^[19]^ Additionally, Yeung et al found that two patients (8.3%) in the inferior graft group had minor localized donor site scarring.^[13]^ The results of the study by Kazancı et al—suggesting a mean difference of 0.09 before the operation and 0.05 after the operation in BCVA between the two groups of superior conjunctival autograft and inferior conjunctival autograft—are consistent with the significant improvement in BCVA that was seen in both of our study's groups.^[27]^

The strengths of the present study included the investigation of a subject that has been scarcely addressed in the current literature with a controlled, parallel-group, single-blind, and randomized method. Also, we evaluated multiple outcomes, including recurrence, inflammation, BCVA, and postoperative complications (including eye burning) in our study. However, there were also certain limitations in this study. First, we included patients from an eye center in one city; therefore, our results may not be applied to the general population, and the study could benefit from a larger sample size that can be considered in future projects. Second, we limited the effect of confounders by proper randomization, but we cannot claim that we have omitted their effect completely. Important factors that could lead to pterygium recurrence, such as smoking and pollution related to work exposure and the environment, were not considered in the allocation of participants in this study. It will be better if these factors are also similar between groups in future studies.

In summary, the recurrence and complication rates of using the IRCF for pterygium surgery were similar to those of the SRCF, suggesting that the inferior conjunctiva is a safe and effective site for rotational flap creation using primary pterygium surgery. It can be used as an alternative to the more common SRCF technique when there is insufficient conjunctiva at the superior bulbar conjunctival region, or it should be saved for future glaucoma surgery. Further multicenter prospective trials with a larger sample size can confirm the benefits of this technique.

##  Acknowledgements 

The authors sincerely thank Mrs. Shila Kianmehr for her expert work on this project at the Research Center.

##  Financial Support and Sponsorship

None.

##  Conflicts of Interest

None.
